# A Case of Acute Compartment Syndrome Resolved Without Surgical Intervention

**DOI:** 10.7759/cureus.26761

**Published:** 2022-07-11

**Authors:** Abdulqader A Alssaggaf, Rakan A Alzahrani, Abdulrahman S Alquzi, Mohammed S Alghamdi, Amal A Alhamdan

**Affiliations:** 1 College of Medicine, King Saud Bin Abdulaziz University for Health Sciences, Jeddah, SAU; 2 Vascular Surgery, King Abdulaziz Medical City, Jeddah, SAU

**Keywords:** idiopathic, diabetic, anticoagulant, conservative management, acute compartment syndrome

## Abstract

Acute compartment syndrome (ACS) is often a result of a traumatic event or fractures. Nevertheless, other non-traumatic etiologies may rarely cause ACS. We present a case of a male patient who presented with unilateral below-the-knee pain without trauma or any external factor and was treated conservatively. A diagnosis of idiopathic compartment syndrome was made by MRI.

## Introduction

Anterior compartment syndrome (ACS) is a limb-threatening and sometimes life-threatening emergency caused by increased pressure in the anterior compartment muscles, that may, in due course, cause hypoxemia [1]. A decrease in tissue perfusion results in bad outcomes like functional impairment, loss of limb, and death in extreme cases [1]. Generally, ACS occurs after traumatic events. Other occurrences that can lead to ACS are ischemia perfusion injury, hemorrhage, phlegmasia cerulea dolens, vascular procedures in patients with a history of bleeding disorders, injections through intravenous or arterial access, soft tissue injury caused by long compression like in lithotomy position, constricting casts or wraps, burns, and iatrogenic causes [1,2]. 

The primary indicators of ACS are pain on a passive stretch that is out of proportion to the injury, swelling, abnormal sensation, and paralysis [3]. However, not all of these symptoms may surface with ACS [4]. Some clinical signs like pallor, reduced capillary refill, and absent peripheral pulses are late signs of ACS [3]. ACS is a surgical emergency that requires immediate intervention to decrease pressure and prevent further complications [5]. 

ACS is seldom recorded to occur without any previous trauma or any other familiar risk factors that cause it [6]. In this case, a 73-year-old male presented with left acute anterior compartment syndrome without identifiable risk factors. The patient’s refusal of surgical intervention and conservative management with noticeable improvement makes this case unique. 

## Case presentation

A 73-year-old male presented to the emergency department with left-sided below-knee pain that started one day before the presentation. The pain began as he was going up the stairs and improved with rest. He denied having any history of trauma, stings, insect bites, or fever. Additionally, he was diabetic and hypertensive, and was on metformin and valsartan, respectively. He was married and a retired soldier with no previous surgeries or notable family history. 

**Figure 1:**
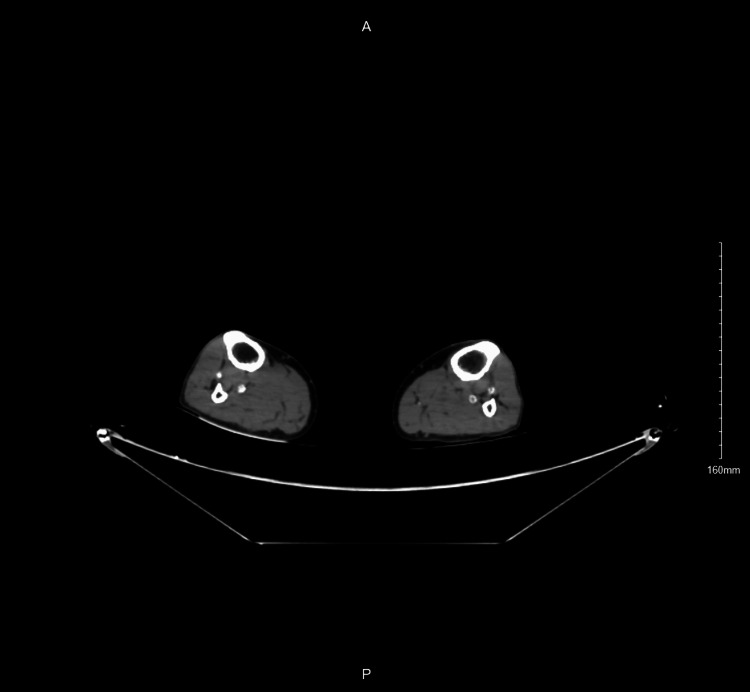
CT angiography shows thrombosis in the anterior tibial artery in the left lower limb.

**Figure 2:**
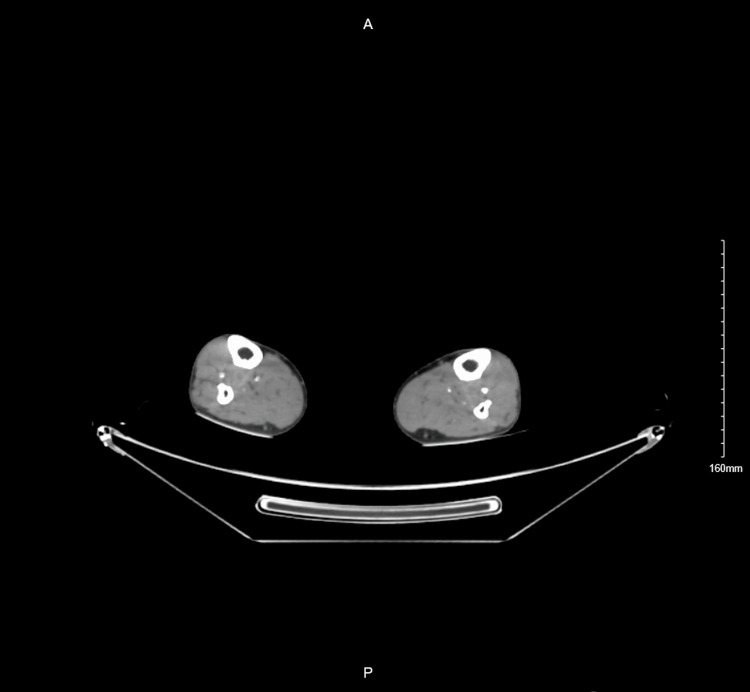
CT angiography shows thrombosis in the peroneal artery in the left lower limb.

On examination in the emergency department, he was vitally stable with a blood pressure of 159/67, heart rate of 90 beats per minute, respiratory rate of 20 breaths per minute, and normal temperature of 36.5 degrees Celsius. The patient was incapable of walking because of pain; however, motor power and sensation were intact in the left leg, with mild coldness. Lower limbs were symmetrically equal in size, with delayed left lower limb capillary refill. There was no palpable pulse or Doppler signal in the dorsalis pedis and posterior tibial arteries of the left limb, but the popliteal artery and femoral artery were palpable. A computed tomography angiography (CTA) revealed thrombosis of the anterior tibial artery and proximal part of the peroneal artery (Figures 1-2). 

**Figure 3:**
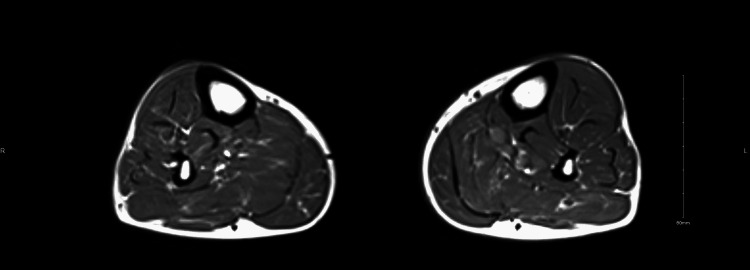
Clear swelling in the anterior compartment of the left calf compared to the right calf. No muscle atrophy or fluid collection can be seen.

The patient was admitted for acute left lower limb ischemia. He was started on intravenous (IV) heparin infusion. The probability of thrombectomy or catheter-directed tissue plasminogen activator (tPA), the risk of distal showering, the possibility of aggravation, and the need for amputation were explained to the patient. However, he decided to continue on heparin as he improved. On the third day of admission, he complained of pain out of proportion in the left leg, developed a fever of 38 degrees Celsius, and developed redness and mild tenderness all over the left leg with no underlying collection. There was crepitus, and the sensation was intact along with biphasic signals in the left anterior tibial artery. An MRI was done, which showed moderate edema related to swelling that involved the left tibialis anterior, extensor digitorum longus, anterior peroneus longus, and medial and lateral gastrocnemius (Figure 3).

**Figure 4:**
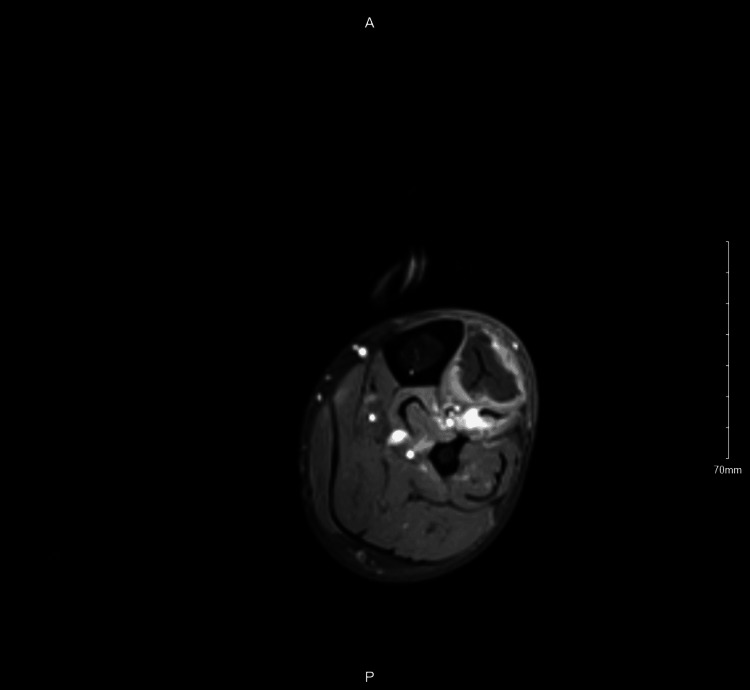
MRI of the anterior compartment of the left lower limb showing tibialis anterior mildly enlarged, and it appears hypointense.

No muscle atrophy, fluid collection, or abnormal bone marrow signal was present. Additionally, laboratory investigations revealed highly elevated creatine phosphokinase which was 348 mcg/L. The options for revascularization were discussed again; however, the patient chose medical management. Consequently, he continued on heparin and IV antibiotics. The symptoms got better with conservative management. After six days, another MRI was done, which confirmed the diagnosis of ACS (Figure 4). 

The patient's condition improved substantially. The pain almost disappeared, the redness started to fade away, and he could move with minimal pain. The patient was discharged with a prescription of warfarin and aspirin. He was advised to avoid trauma, encourage mobilization, and close follow-ups. After one month, the patient came to the clinic walking and pain-free. 

## Discussion

Acute compartment syndrome is defined as a rise in intra-compartmental hydrostatic pressure in the limb, leading to decreased tissue perfusion. It is a surgical emergency that requires timely intervention. Though it was described more than 100 years ago, it is still difficult to diagnose. Delay in diagnosis and treatment can result in life-changing complications such as tissue necrosis, limb loss, or death. Commonly ACS appears after a traumatic event. Contrarily, non-traumatic causes like nephrotic syndrome, diabetes mellitus, leukemia, bleeding disorders, warfarin and heparin use, viral-induced myositis, and rhabdomyolysis rarely lead to ACS. Our patient has type 2 diabetes mellitus on metformin with the last HbA1C level of 8.3% [7,8].

There are several theories concerning how diabetes can lead to this condition. One of the hypotheses is muscle infarction because of microangiopathic disease, which causes edema secondary to the infraction [9]. Another hypothesis proposes that it is linked to hyperglycemia and osmotic fluid collection, which can cause increased pressure in the compartment [10]. In our case, the patient denied having any history of diabetic emergencies like hyperosmolar hyperglycemic state (HHS). 

The diagnosis of ACS depends mainly on physical examination and the major symptoms of the patients. These symptoms are the 6 P's: pain, paraesthesia, pulselessness, paralysis, pallor, and poikilothermia. Measuring intra-compartmental pressure (ICP) is beneficial but not required to diagnose ACS. These symptoms, along with ICP measurement and laboratory findings such as serum creatine phosphokinase and urine myoglobin are used to evaluate the development of the condition. Not all patients exhibit the common presentation of ACS. Therefore, physicians should pay attention to and have a high suspicion index for unusual cases [4,11]. Our patient did not have the traditional picture of ACS, so the diagnosis was delayed. The diagnosis was done based on the rapid progression of symptoms and signs over a few hours. Notable findings include pain out of proportion and pain with passive stretch of muscles. Creatine phosphokinase was increased. Also, MRI showed increased signal intensity in the entire anterior compartment.

Fasciotomy is the most useful treatment for ACS, incising skin and muscle fascia to decrease intra-compartment pressure [12]. We were unable to find a case of conservatively treated leg ACS; however, Riede U et al. reported a conservatively managed case of thigh ACS. The patient had suffered high-energy sports trauma to the thigh as opposed to our patient, who developed the symptoms suddenly while going up the stairs. In the case of Riede U et al., daily subcutaneous enoxaparin was administered for four weeks prophylactically for deep venous thrombosis. Bromelain for the swelling and paracetamol for the pain was also given. Physical therapy focusing on active and passive range of motion started two days after admission. Their patient's condition improved, and he was discharged on the fifth day of admission [13]. Our patient started on IV heparin infusion with an international normalized ratio (INR)goal of 2-3, paracetamol, and morphine for the pain. He encountered severe pain and fever on the third day of admission. Consequently, vancomycin and meropenem were included. On the seventh day, the diagnosis of ACS was established by an MRI. The patient chose to continue with the same plan as he showed improvement. On the tenth day, he was discharged and was prescribed warfarin, aspirin, amoxicillin/clavulanate acid, and celecoxib.

## Conclusions

Acute compartment syndrome is regarded as a surgical emergency. Sometimes the clinical diagnosis can be difficult, and a high suspicion index is needed in atypical presentations. We report a conservatively managed case of ACS. This could explain how to deal with ACS when surgical intervention is not an alternative.
